# Analysis of serum interleukin(IL)‐1α, IL‐1β and IL‐18 in patients with systemic sclerosis

**DOI:** 10.1002/cti2.1045

**Published:** 2019-04-06

**Authors:** Emily Lin, Fabien B Vincent, Joanne Sahhar, Gene‐Siew Ngian, Rangi Kandane‐Rathnayake, Rachel Mende, Eric F Morand, Tali Lang, James Harris

**Affiliations:** ^1^ Rheumatology Group Centre for Inflammatory Diseases School of Clinical Sciences at Monash Health Monash University Clayton VIC Australia; ^2^ Department of Rheumatology Monash Health & Monash University Clayton VIC Australia; ^3^ Present address: Tali Lang, The Szalmuk Family Department of Medical Oncology Cabrini Institute Malvern VIC 3144 Australia

**Keywords:** biomarker, IL‐18, IL‐1β, inflammation, interleukin (IL)‐1α, scleroderma

## Abstract

**Objectives:**

Systemic sclerosis (SSc) is an autoimmune disease characterised by fibrosis, vascular dysfunction and immune dysregulation. The pathogenesis of SSc remains poorly understood, although studies have indicated a role for the innate immune response.

**Methods:**

Here, we measured serum interleukin (IL)‐1α, IL‐1β and IL‐18 levels in 105 SSc patients and 47 healthy controls (HC) and analysed them with respect to multiple clinical parameters.

**Results:**

Serum IL‐18 concentrations were significantly higher in SSc patients than in HC, while no significant differences in concentrations of IL‐1α and IL‐1β were observed between SSc and HC. In both SSc and HC serum, IL‐1α and IL‐1β were positively correlated, while in SSc, both cytokines negatively correlated with IL‐18. Serum IL‐18 was significantly negatively correlated with both carbon monoxide transfer coefficient (KCO) and diffusing capacity of the lungs for carbon monoxide (DLCO). Serum IL‐1β was positively correlated with the modified Rodnan skin score (mRSS), particularly in patients with limited subtype. DLCO, KCO and tricuspid regurgitation (TR) velocity were significantly higher in patients with high serum IL‐1β. Serum IL‐1α was significantly lower in SSc patients with low KCO and positively correlated with KCO. SSc patients with high serum IL‐1α concentrations were more likely to have digital ulcers.

**Conclusions:**

Our data suggest that these IL‐1 family cytokines may have different roles in the pathogenesis of SSc fibrotic complications.

## Introduction

Systemic sclerosis (SSc, scleroderma) is a chronic systemic autoimmune disease characterised by fibrosis, vascular dysfunction and immune dysregulation.^1^ Although genetic and environmental factors have been implicated in the development of SSc, its pathogenesis remains poorly understood.^2^ Increasing evidence suggests a critical role for the innate immune system in the induction and maintenance of SSc.^3^ In particular, a number of cytokines including interleukin (IL)‐6,^4^ transforming growth factor (TGF)‐β,^5^ macrophage migration inhibitory factor (MIF)^6^ and members of the IL‐1 superfamily^7^ have been reported in the pathogenesis of SSc. However, in contrast to other autoimmune diseases, such as rheumatoid arthritis, where biologic therapies targeting cytokines involved in disease pathogenesis have transformed disease management,^8^ a clear cytokine profile has not yet been defined in SSc to facilitate advances in targeted therapy.

The IL‐1 superfamily of cytokines encompasses both pro‐ and anti‐inflammatory members, and includes IL‐1α, IL‐1β, IL‐18, IL‐33, IL‐37 and IL‐38. IL‐1α and IL‐1β are both pro‐inflammatory, pyrogenic cytokines released by numerous cells, particularly innate immune cells such as monocytes, macrophages and dendritic cells.^9^ They share common properties in their regulation and expression, as well as a common receptor, IL‐1R1, and both induce inflammatory responses via induction of cyclooxygenase type‐2 (COX‐2), type 2 phospholipase A and inducible nitric oxide synthase (iNOS).^9^ Moreover, in concert with IL‐23, IL‐1α and IL‐1β can drive IL‐17 release by T cells.^10^ IL‐18, while structurally homologous to IL‐1α and IL‐1β, binds a different receptor, IL‐18R, and has distinct functions; in combination with IL‐12, it drives Th1‐mediated responses, including the release of interferon (IFN)‐γ.^10^

Previous studies have shown that peripheral blood mononuclear cells (PBMC) and fibroblasts from SSc patients produce more IL‐1α compared to those from healthy controls (HC).^11^, ^12^, ^13^ Moreover, carriers of the IL‐1α (‐889) polymorphism, which predisposes patients to increased IL‐1α production, are more susceptible to SSc.^14^ To date, two small studies have shown significant elevation of serum IL‐1α in SSc patients compared to HC.^15^, ^16^ One of these studies analysed associations between IL‐1α and clinical SSc phenotypes, reporting an association with finger contractures.^16^

IL‐1β is implicated in the pathogenesis of SSc through its role in fibrosis. Studies have demonstrated that IL‐1β is an important cytokine involved in a mouse model of bleomycin‐induced pulmonary fibrosis (BIPF).^17^, ^18^ Similarly to IL‐1α, SSc PBMC and fibroblasts produce more IL‐1β compared to those from HC.^17^, ^19^ Serum IL‐1β has also been reported to be higher in SSc than in HC.^20^ Some studies have shown correlations between IL‐1β and SSc clinical parameters, such as a negative correlation between IL‐1β measured in bronchoalveolar lavage fluid with the forced vital capacity (FVC), and a positive correlation between skin gene expression of IL‐1β and the modified Rodnan skin score (mRSS).^20^, ^21^, ^22^

Involvement of IL‐18 in the pathogenesis of SSc is an ongoing matter of debate, with studies reporting conflicting results.^23^ *In vitro*, similar to IL‐1α and IL‐1β, increased IL‐18 secretion has been observed in PBMC and fibroblasts from SSc patients compared to HC.^4^, ^17^ As opposed to IL‐1β, IL‐18 was shown to have anti‐fibrotic functions mediated through the extracellular signal‐regulated kinase pathway in human SSc dermal fibroblasts.^24^ However, increased skin expression of IL‐18 was observed in SSc patients compared to HC and found to be positively correlated with the mRSS,^22^ while studies in murine models of BIPF suggest a causative role for IL‐18 in skin thickness and pulmonary fibrosis.^17^ While no significant differences in serum concentrations of IL‐18 have been reported in SSc patients compared to HC, patients with renal involvement had decreased PBMC‐produced IL‐18.^4^

One previous study has looked at IL‐1α, IL‐1β and IL‐18 (as well as the IL‐1 family cytokine IL‐33) in a relatively modest‐sized Chinese cohort (56 patients and 56 HC) and found that only serum IL‐1β and IL‐33 were higher in SSc in multivariable analysis. No clinical associations with any of these cytokines were found.^25^ Here, we examined the clinical relevance of serum IL‐1α, IL‐1β and IL‐18 in a large, well‐characterised and prospectively followed SSc cohort. We found that serum IL‐18, but not IL‐1α or IL‐1β, was significantly higher in SSc patients than in HC. Moreover, we observed significant correlation between serum IL‐1β and IL‐1α with mRSS score and anti‐topoisomerase I antibody (Ab), respectively, suggesting a potential role of these cytokines in SSc fibrotic complications.

## Results

### Participant characteristics

In all, 105 SSc patients were enrolled in this study, and their demographics and disease characteristics are outlined in Tables 1 and 2. Briefly, the mean (SD) age and median [IQR] disease duration were 60.1 (13.9) and 12.3 [6.8, 19.3] years, respectively. Most patients were female (82.9%) and of Caucasian ethnicity (83.5%). The median [IQR] mRSS was 5 [3, 8], and 22% of patients had diffuse SSc subtype. Of the 47 HC who participated in this study with a median [IQR] age of 37.6 [29.1, 45.9] years, 72% were female, and 70% were of Caucasian ethnicity. Although HC were gender‐ and ethnicity‐matched to the SSc cohort, there was a statistically significant difference in age between the two cohorts (Table 1).

**Table 1:** Demographics and serum IL‐1 family cytokines in SSc and HC

	HC (*N* = 47)	SSc (*N* = 105)	*P*‐value
Demographics			
Age (years), *mean* (SD)	37.6 (10.6)	60.1 (13.9)	< 0.01
Female, *n* (%)	34 (72.3%)	87 (82.9%)	0.14
Ethnicity^a^, *n* (%)			
Caucasian	33 (70.2%)	86 (83.5%)	0.09
Asian	11 (23.4%)	10 (9.7%)	
Other	3 (6.4%)	7 (6.8%)	
Serum cytokines			
Detectable IL‐1α*, n* (%)	35 (74.5%)	78 (74.3%)	0.98
IL‐1α (pg mL^−1^), *median* [IQR]	3 [2, 41]^b^	11 [2, 29]^c^	0.51
Detectable IL‐1β*, n* (%)	26 (55.3%)	68 (64.8%)	0.27
IL‐1β (pg mL^−1^), *median* [IQR]	5 [1, 16]	7 [1, 17]	0.37
Detectable IL‐18*, n* (%)	47 (100%)	103 (98.1%)	0.9
IL‐18 (pg mL^−1^), *median* [IQR]	183 [135, 258]	265 [183, 362]^d^	< 0.01

HC, healthy controls; IL, interleukin; SSc, systemic sclerosis.

a Two missing values in the SSc cohort.

b *N* = 42; When including 5 HC with serum IL‐1α concentrations higher than uLOD (*N* = 47): median [IQR] serum IL‐1α concentrations 19 [2, 70]; *P *=* *0.9.

c *N* = 87; When including 18 SSc patients with serum IL‐1α concentrations higher than uLOD (*N* = 105): median [IQR] serum IL‐1α concentrations 17 [2, 87]; *P *=* *0.9.

d *N* = 103; When including 2 SSc patients with serum IL‐18 concentrations higher than uLOD (*N* = 105): median [IQR] serum IL‐18 concentrations 266 [191, 362]; *P *<* *0.01.

**Table 2:** Disease characteristics of SSc patients

	SSc patients (*N* = 105)
Clinical parameters	
Disease duration (years), *median* [IQR] (range)	12.3 [6.8, 19.3] (0.6, 46.7)
Diffuse SSc, *n* (%)	23 (21.9%)
Clinical manifestation	
PAH*, n* (%)	5 (4.8%)
Pericardial effusion*, n* (%)	5 (4.8%)
ILD*, n* (%)	35 (33.3%)
Systemic hypertension^a^, *n* (%)	32 (32%)
Renal crisis*, n* (%)	4 (3.8%)
Digital ulcers^a^ *, n* (%)	14 (14%)
mRSS^a^, *median* [IQR] (range)	5 [3, 8] (0, 20)
mRSS > 18*, n* (%)	1 (1%)
GAVE, *n* (%)	9 (8.6%)
Reflux oesophagitis*, n* (%)	59 (56.2%)
Oesophageal stricture*, n* (%)	9 (8.6%)
Oesophageal dysmotility*, n* (%)	5 (4.8%)
RP^a^ *, n* (%)	85 (85%)
Calcinosis^a^ *, n* (%)	23 (23%)
Myositis*, n* (%)	2 (1.9%)
Synovitis^a^ *, n* (%)	11 (11%)
Joint contracture^a^, *n* (%)	27 (27%)
Pulmonary and cardiac function tests	
FVC (% predicted)^a^, mean (SD)	93.7 (18.2)
FEV1 (% predicted)^a^, mean (SD)	89.7 (18.3)
DLCO (% predicted)† ^,^ b, median [IQR] (range)	59.5 [48.1, 73.6] (24.6, 116.4)
KCO (% predicted)‡ ^,^ c, mean (SD)	64.4 (17.2)
6‐min walk distance (m)^d^, median [IQR] (range)	508 [432, 560] (252, 697)
LVEF (%)^e^, median [IQR] (range)	65 [60, 65] (35, 75)
sPAP (mmHg)^e^ *,* median [IQR] (range)	31 [28, 39] (21, 108)
Clinical laboratory data	
ANA +ve^a^ *, n* (%)	100 (96.2%)
ANA anti‐centromere +ve^a^ *, n* (%)	42 (40.4%)
Anti‐topoisomerase I^a^ *, n* (%)	25 (24.3%)
Anti‐RNA polymerase III +ve^a^ *, n* (%)	9 (8.8%)
ANCA +ve^a^, *n* (%)	27 (26.5%)
MPO specificity*, n* (%)	3 (2.9%)
PR‐3 specificity*, n* (%)	3 (2.9%)
CRP (mg L^−1^)^b^, median [IQR] (range)	3.5 [1.4, 6] (0.2, 46)
ESR (mm h^−1^)^f^, median [IQR] (range)	10 [5, 17] (1, 77)
Creatinine (μmol L^−1^)^g^, median [IQR] (range)	65 [54, 76] (36, 149)
Treatment, *n* (%)	
Glucocorticoids	24 (22.9%)
PDE5 inhibitor	5 (4.8%)
ERA	5 (4.8%)
Ca2^+^ channel antagonist	51 (48.6%)
Anticoagulant	7 (6.7%)
Anti‐platelet agent	19 (18.1%)
ACE inhibitor	11 (10.5%)
Angiotensin II receptor blockers	17 (16.2%)
Beta blockers	5 (4.8%)

ANA, antinuclear antibodies; ANCA, anti‐neutrophil cytoplasmic antibodies; CRP, C‐reactive protein; DLCO, Hb‐ and gender‐corrected diffusing capacity of the lungs for carbon monoxide; ERA, endothelin receptor antagonist; ESR, erythrocyte sedimentation rate; FEV1, forced expiratory volume in one‐second; FVC, forced vital capacity; GAVE, gastric antral vascular ectasia; ILD, interstitial lung disease; KCO, carbon monoxide transfer coefficient; LV, left ventricular; LVEF, left ventricular ejection fraction; MPO, myeloperoxidase; mRSS, modified Rodnan skin score; PAH, pulmonary arterial hypertension; PDE5, phosphodiesterase 5; PR‐3, proteinase 3; RP, Raynaud's phenomenon; RV, right ventricular; Sm, Smith; sPAP, systolic pulmonary arterial pressure; SSc, systemic sclerosis.

^a^≤ 5 missing values; ^b^10 missing values; ^c^7 missing values; ^d^76 missing values; ^e^28 missing values; ^f^12 missing values; ^g^8 missing values.

* Corrected for haemoglobin and gender.

‡ DLCO corrected for lung volume.

### Serum IL‐1 family cytokines in SSc

**Figure 1:**
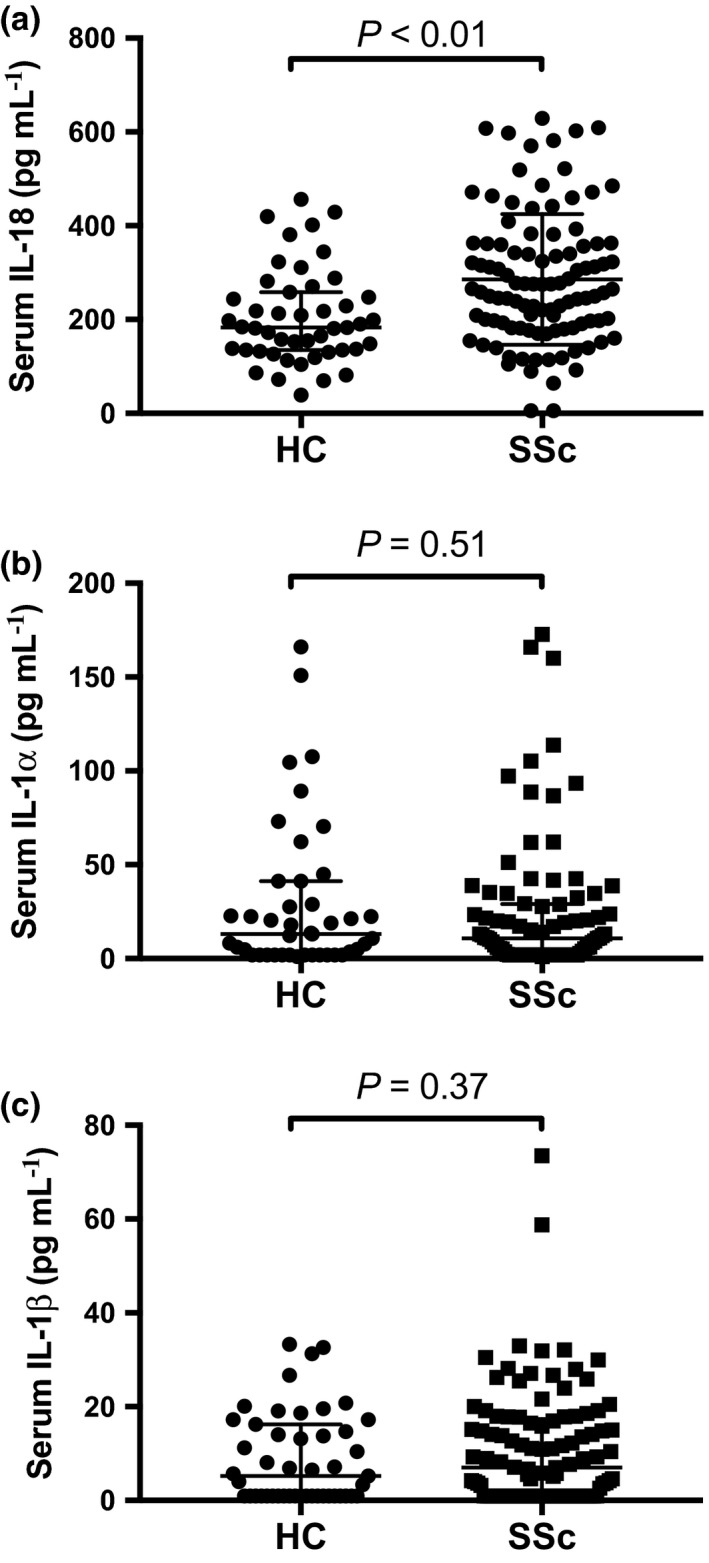
Serum IL‐1α, IL‐1β and IL‐18 concentrations in SSc and HC. **(a)** Serum IL‐18 concentrations in HC (*N* = 47) vs. SSc patients (*N* = 103). **(b)** Serum IL‐1α concentrations in HC (*N* = 42) vs. SSc patients (*N* = 87). **(c)** Serum IL‐1β concentrations in HC (*N* = 47) vs. SSc patients (*N* = 105). Horizontal bars indicate medians, and corresponding error bars indicate IQR; a Wilcoxon rank‐sum test was used to examine differences between two groups. All samples were run in duplicate.

IL‐18 was detectable in serum samples from all 47 HC and 98% (103/105) of SSc patients. Similarly, serum IL‐1α was detectable in 75% (35/47) of HC and 74% (78/105) of SSc patients, and IL‐1β was detectable in 55% (26/47) of HC and 65% of (68/105) of SSc patients. Hence, the proportion of detectability of these serum cytokines were similar in HC and SSc groups (Table 1). Serum IL‐18 concentrations were statistically significantly higher in SSc patients than in HC (Figure 1a), confirmed by linear regression analyses after adjusting for age (adjusted ratio of GM 1.28; 95% CI 1.02, 1.6; *P *=* *0.03). However, serum concentrations of IL‐1α and IL‐1β were not significantly different in SSc compared to HC (Figure 1b–c).

We found a statistically significant and strong positive correlation between serum concentrations of IL‐1α and IL‐1β in SSc and more moderate positive correlation in HC (Supplementary figure 1a–b). Serum concentrations of both IL‐1α and IL‐1β were negatively correlated with IL‐18 in SSc, but not in HC (Supplementary figure 1c–f).

### Serum IL‐18 and SSc clinical parameters

**Figure 2:**
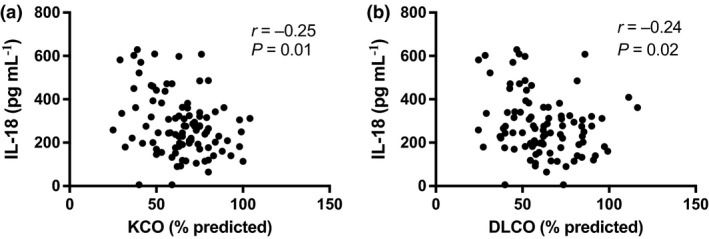
Serum IL‐18 and SSc clinical parameters. **(a)** Correlation between serum concentrations of IL‐18 and KCO (% predicted) in SSc (*N* = 96). **(b)** Correlation between serum concentrations of IL‐18 and DLCO (% predicted) in SSc (*N* = 93). A Spearman's correlation test was used to examine the correlation between two continuous variables.

We next examined differences in serum IL‐18 concentrations according to SSc demographics and clinical parameters. No significant correlation was observed between serum IL‐18 and disease duration (*r* = −0.05; *P *=* *0.65; *N* = 103) or mRSS score (*r* = −0.13; *P *=* *0.2; *N* = 98). No significant difference in serum IL‐18 concentrations was observed between patients with diffuse and limited subtype (Supplementary table 1). Serum IL‐18 concentrations were moderately negatively correlated with KCO (Figure 2a), a finding restricted to patients with limited SSc (*r* = −0.23; *P *=* *0.05; *n* = 74). Accordingly, there was a nonsignificant trend towards increased serum IL‐18 concentrations in SSc patients with low KCO (Supplementary table 2). In line with this negative correlation between serum IL‐18 concentrations and KCO, we found that serum IL‐18 concentrations were also moderately negatively correlated with DLCO in the whole cohort (Figure 2b), as well as being restricted to diffuse SSc patients (*r* = −0.48; *P *=* *0.02; *n* = 22). However, no significant differences in serum IL‐18 concentrations were observed according to the presence of ILD, PAH, low FVC or low DLCO (Supplementary tables 1 & 2). Serum IL‐18 concentrations were statistically significantly higher in SSc patients with high serum creatinine than in those without (Supplementary table 3). No significant difference in serum IL‐18 concentrations was observed when examining any other SSc clinical parameters (Supplementary tables 1–4).

We also stratified the SSc cohort into low and high serum IL‐18 subsets, using the median IL‐18 concentration (264.8 pg mL^−1^) as a cut‐off. Serum creatinine concentrations were statistically significantly higher in SSc patients with high serum IL‐18 than in those with low serum IL‐18 (Supplementary table 5). No significant differences were observed in other SSc patient demographics or disease characteristics according to high/low serum IL‐18 (Supplementary table 5).

### Serum IL‐1β and SSc clinical parameters

**Figure 3:**
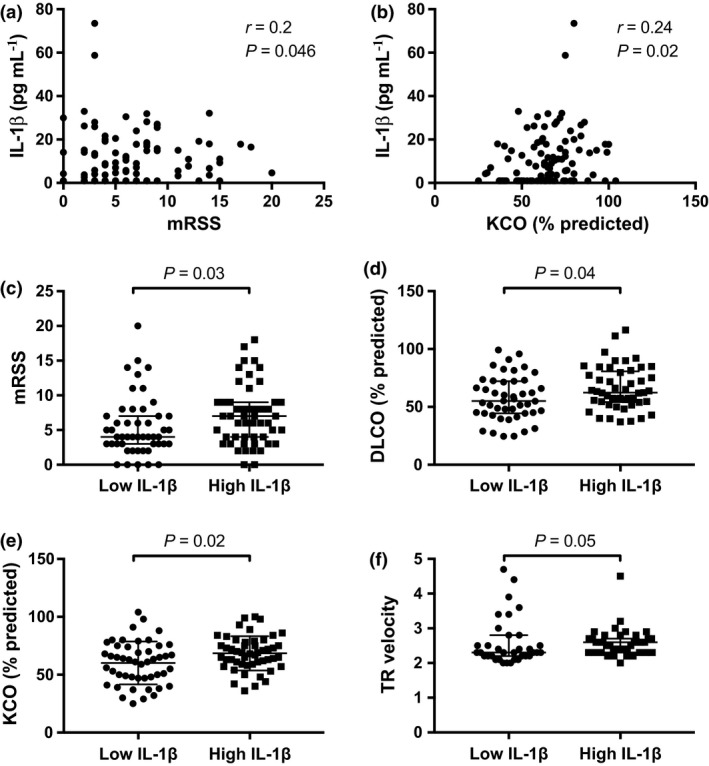
Serum IL‐1β and SSc clinical parameters. Correlation between serum concentrations of IL‐1β and **(a)** mRSS (*N* = 100) and **(b)** KCO (% predicted) (*N* = 98) in SSc. **(c)** mRSS index, **(d)** DLCO (%), **(e)** KCO (% predicted) and **(f)** TR velocity in patients with low (*n* = 53) or high (*n* = 52) serum IL‐1β concentrations. **a** and **b:** Spearman's correlation test was used to examine the correlation between two continuous variables. **c–f:** Horizontal bars indicate medians, and corresponding error bars indicate IQR; a Wilcoxon rank‐sum test was used to examine differences between two groups.

We next examined differences in serum IL‐1β concentrations according to SSc demographics and clinical parameters. Serum IL‐1β concentrations were statistically significantly positively correlated with mRSS score in the whole cohort (Figure 3a) and in patients with limited subtype (*r* = 0.24; *P *=* *0.03; *n* = 77). However, no significant difference in serum IL‐1β concentrations was observed between patients with diffuse and limited subtype (Supplementary table 1). Serum IL‐1β concentrations were also statistically significantly correlated with the right finger–palm distance (*r* = 0.21; *P *=* *0.04; *n* = 98) and were significantly increased in patients with joint contracture compared to those without (Supplementary table 1). No correlation was observed between serum IL‐1β and disease duration (*r* = 0.04; *P *=* *0.68; *N* = 105). Serum IL‐1β concentrations were moderately positively correlated with KCO (Figure 3b), a finding restricted to patients with limited disease (*r* = 0.28; *P* = 0.02; *n* = 76). In line with this, we found a significant positive correlation between serum IL‐1β concentrations and DLCO in patients with limited disease (*r* = 0.23; *P *=* *0.05; *n* = 73). Serum IL‐1β concentration was significantly increased in patients with ILD, however, only in those with diffuse SSc subtype (*n* = 12 vs *n* = 11; 11 [7, 16] vs 4 [1, 7] pg mL^−1^; *P *=* *0.03). Moreover, a higher proportion of patients with ILD were observed in the high serum IL‐1β subset, with borderline significance (*P *=* *0.07) (Supplementary table 6). No significant differences in serum IL‐1β concentrations were observed when examining any other SSc clinical parameters (Supplementary tables 1–4).

Upon stratification of the SSc cohort into low and high serum IL‐1β subsets according to median IL‐1β concentration (7.1 pg mL^−1^), we observed that mRSS was statistically significantly higher in patients with high serum IL‐1β than in those without (Figure 3c and Supplementary table 6). DLCO and KCO, as well as TR velocity on TTE, were statistically significantly higher in patients with high serum IL‐1β than in those without (Figure 3d–f and Supplementary table 6). However, no statistically significant differences were observed between patient subsets with high or low serum IL‐1β regarding ILD, PAH, FVC, or other SSc patient demographics or disease characteristics (Supplementary table 6).

### Serum IL‐1α and SSc clinical parameters

**Figure 4:**
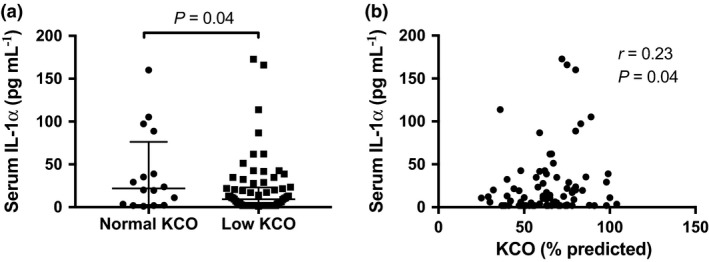
Serum IL‐1α and SSc clinical parameters. **(a)** Serum IL‐1α concentrations in patients with normal (*n* = 15) vs. low (*n* = 67) KCO (% predicted). **(b)** Correlation between serum IL‐1α concentration and KCO (% predicted) in SSc (*N* = 82). **a**: Horizontal bars indicate medians, and corresponding error bars indicate IQR; a Wilcoxon rank‐sum test was used to examine differences between two groups. **b**: A Spearman's correlation test was used to examine the correlation between two continuous variables.

We next examined differences in serum IL‐1α concentrations according to SSc demographics and clinical parameters. No significant difference in serum IL‐1α concentrations was observed between patients with diffuse and limited subtype (Supplementary table 1). No correlation was seen between serum IL‐1α and mRSS score (*r* = 0.07; *P *=* *0.56; *N* = 82) or disease duration (*r* = −0.11; *P *=* *0.29; *N* = 87). Serum IL‐1α concentrations were statistically significantly lower in SSc patients with high serum creatinine (Supplementary table 3) and in SSc patients with low KCO than in those without (Figure 4a and Supplementary table 2), and serum IL‐1α concentrations were moderately positively correlated with KCO (Figure 4b). In line with these data, we found significant positive correlations between serum IL‐1α and KCO in patients with limited (*r* = 0.27; *P *=* *0.03; *n* = 66) but not diffuse disease subtype. No significant differences in serum IL‐1α concentrations were observed when examining any other SSc clinical parameters (Supplementary tables 1–4).

When stratifying patients into high and low serum IL‐1α subsets, using the median IL‐1α concentration (17.1 pg mL^−1^) as a cut‐off, we observed a higher proportion of SSc patients with digital ulcers in the high serum IL‐1α subset than in the low serum IL‐1α subset (Supplementary table 7). No differences were observed in other SSc patient demographics or disease characteristics between the high and low serum IL‐1α subsets (Supplementary table 7).

## Discussion

The IL‐1 superfamily members IL‐1α, IL‐1β and IL‐18 are acknowledged to play a role in the pathogenesis of fibrosis and autoimmune disease, including SSc.^7^ Most SSc studies, however, have been conducted in small and poorly described populations.^16^, ^20^, ^25^ To date, our study is the largest and most ethnically diverse to investigate the clinical relevance of serum IL‐1α, IL‐1β and IL‐18 in SSc. We observed that SSc patients have significantly higher levels of IL‐18, but not IL‐1α or IL‐1β, than in HC. Correlation between serum IL‐1β and mRSS score suggests a potential role of this cytokine in SSc fibrotic complications.

Our finding of higher levels of serum IL‐18 in SSc patients than in HC is in line with a previously published study.^25^ We report, for the first time, negative correlations between serum IL‐18 and both KCO and DLCO. Although no significant associations were noted in relation to low FVC or presence of ILD or PAH, this finding might implicate IL‐18 in SSc‐related lung disease.^23^ To the best of our knowledge, no prior published study has suggested a role for IL‐18 in SSc pulmonary disease. Our findings that patients with high IL‐18 have significantly higher serum creatinine are of interest. No studies have investigated serum IL‐18 levels in SRC, although our SRC subset is too small to draw any solid conclusions. One study has shown that PBMC from SSc patients with renal involvement produced less IL‐18 in response to stimulation with phytohaemagglutinin (PHA) compared to those without renal involvement.^4^ However, studies in other autoimmune conditions, such as SLE, strongly suggest a role for IL‐18 in renal disease.^26^, ^27^, ^28^ Future research examining the potential role of IL‐18 in renal, cardiac and pulmonary SSc manifestations on a larger prospective cohort would be worthwhile. Likewise, further studies investigating the molecular and cellular mechanisms by which IL‐18 might regulate fibroblasts and fibrosis would be of considerable interest.

In contrast with previous studies,^20^, ^25^ we neither observed elevated serum IL‐1β in SSc compared to HC, nor detected differences in IL‐1β levels between lcSSc and dcSSc. These discrepancies may be accounted for by the distinct ethnicities and difference in disease duration across studies. A shorter mean disease duration period described in one of those previous studies^20^ may suggest that differences in IL‐1β are reflective of earlier stages of disease progression. However, in line with a previous study examining gene expression of IL‐1β in SSc skin manifestation,^22^ together with the well‐described role of IL‐1β in fibrosis,^18^, ^29^ we did observe a positive correlation between serum IL‐1β concentration and the mRSS index, particularly in patients with limited subtype. Moreover, serum IL‐1β was positively correlated with other parameters of increased skin thickening, particularly the finger–palm distance and the presence of joint contractures, further supporting a role of IL‐1β in SSc fibrotic complications. However, in contrast to these findings regarding IL‐1β in the skin, we observed that serum IL‐1β was positively correlated with KCO, and patients with high serum IL‐1β had higher DLCO and KCO, suggesting a reduced risk of lung fibrosis and PAH. Contrary to this finding, IL‐1β was positively correlated with increased TR velocity, which is usually associated with an increased risk of PAH.^30^, ^31^ In light of these divergent results, and when considering the lack of correlation between serum IL‐1β and the presence of either ILD or PAH, a larger longitudinal study investigating the role of IL‐1β in SSc lung disease would be of considerable value.

We did not observe a significant difference in serum IL‐1α between SSc and HC, in line with some previous studies, although others have reported elevated serum IL‐1α in SSc.^15^, ^16^, ^32^ We report for the first time that SSc patients with high serum IL‐1α concentrations were more likely to have digital ulcers. Similar to our observation with IL‐1β, we found that IL‐1α was positively correlated with KCO. These data emphasise the need for further research examining the role of IL‐1α in SSc pathogenesis, particularly in pulmonary manifestations and obliterative vasculopathy.

While our cohort contained no cases of SSc with clinical features of vasculitis, it is also worth noting that, when compared to previously published studies,^33^, ^34^ the proportion of anti‐neutrophil cytoplasmic antibody (ANCA)‐positive patients in our cohort of 26.5% (27/102) is relatively high; only a few of them, however, are positive on the more specific MPO and PR‐3 auto‐Abs (2.9% each; Table 2), which is lower than or in line with previously published studies.^33^, ^35^

Caveats to the interpretation of our findings apply. Firstly, while being a large and well‐characterised SSc cohort, this is a single‐centre study of patients with prevalent disease. Secondly, the HC cohort was not age‐matched to the SSc cohort; however, a multivariable regression model adjusting for age was applied when appropriate. Moreover, while on the one hand the ethnically varied nature of our large cohort is advantageous, it may also explain the absence of some associations described in previous studies. Finally, some phenotypic subsets, such as SRC and PAH, were too small to enable meaningful analysis.

In conclusion, we report a marked elevation of serum IL‐18, but not IL‐1α and IL‐1β, in SSc patients compared to HC. The observed positive correlation between IL‐1β and parameters of increased skin thickening suggests a potential role for this cytokine in SSc fibrotic complications. These findings underscore the value of further research to investigate the mechanisms through which the IL‐1 superfamily may contribute to SSc fibrotic complications.

## Methods

### Patients and clinical assessments

Between August 2015 and August 2017, patients attending the Scleroderma Clinic at Monash Health were prospectively enrolled in this study. Patients were eligible if they were ≥ 18 years and fulfilled the 2013 American College of Rheumatology (ACR)/European League Against Rheumatism (EULAR) classification criteria for SSc.^36^ These patients were also part of the Australian Scleroderma Cohort Study, a longitudinal study of SSc cardiorespiratory outcomes. Data on organ involvement, drug treatment, routine laboratory markers (creatinine, erythrocyte sedimentation rate (ESR), C‐reactive protein (CRP), estimated glomerular filtration rate), pulmonary function tests (PFT), high‐resolution computed tomography (HRCT) chest, transthoracic echocardiogram (TTE) and right‐heart catheter (RHC) were recorded annually. Other clinical parameters measured were interstitial lung disease (ILD), pulmonary arterial hypertension (PAH), pericardial effusion, FVC, forced expiratory volume in one‐second (FEV1), DLCO (corrected for haemoglobin and gender) and KCO (= DLCO/alveolar volume ratio) as described previously.^37^ Low FVC, FEV1, DLCO and KCO were all defined as < 80% predicted. At TTE, low left ventricular ejection fraction (LVEF) was defined as < 55%, and abnormal systolic PAP (sPAP) was defined as > 40 mmHg. Gastric antral vascular ectasia (GAVE) and reflux oesophagitis were diagnosed by gastroscopy. Scleroderma renal crisis (SRC) was defined as the presence of at least two of new‐onset systemic hypertension, rising creatinine or microangiopathic anaemia. Disease duration was calculated from the date of the first non‐Raynaud's manifestation of SSc.^38^ Patients were classified as either limited (lcSSc) or diffuse (dcSSc) subtype according to the LeRoy criteria.^39^ The mRSS index was used to assess the extent of skin involvement.^40^ Screening results for auto‐Ab were recorded at the initial study visit. When screening for ANCA (cANCA, pANCA, atypical ANCA) was positive using indirect immunofluorescence, sera quantification of anti‐myeloperoxidase (MPO) and anti‐proteinase‐3 (PR‐3) antibodies was subsequently performed by ELISA. Standard indirect immunofluorescence and ELISA tests for ANCA were performed by relevant pathology departments. All patients received standard‐of‐care therapy.

Adult healthy individuals were enrolled as a HC group between February and August 2017. The HC group was gender‐ and ethnicity‐matched to the SSc cohort. Written, informed consent was obtained from all participants. This study was approved by the Human Research Ethics committee of Monash Health and carried out in accordance with the *National Statement of Ethical Conduct in Human Research (2007*).

### Serum cytokine quantification

Venous blood was collected by venepuncture, and serum was separated using serum‐separating tubes, and stored at −80°C until further use, as previously described.^41^ Commercial enzyme‐linked immunosorbent assays (ELISA) kits were used to quantify concentrations of serum IL‐1α (ELISA MAX DeluxeTM kit, BioLegend, CA, USA), IL‐1β (ELISA MAX DeluxeTM kit, BioLegend, CA, USA) and total IL‐18 (DuoSet^®^ ELISA kit, R&D Systems, MN, USA), according to manufacturers’ protocols. Readings below the lower limit of detection were assigned an arbitrary value of half the lowest standard value (IL‐1α, 1.95 pg mL^−1^; IL‐1β, 0.98 pg mL^−1^; IL‐18, 5.86 pg mL^−1^) for statistical analysis. Serum samples with readings above the upper limit of detection (IL‐18: 2 SSc samples; IL‐1α: 18 SSc, 5 HC samples), even after further sample dilutions, were excluded from data analysis.

### Statistical analysis

Statistical analysis was performed using Stata 14.2 (StataCorp, College Station, Texas, USA) and GraphPad (Prism V.7.0d, San Diego, CA, USA) software. Normally distributed variables were reported as mean and standard deviation (SD), and a two sample *t*‐test or ANOVA was used to examine the differences between two or more than two groups, respectively. Non‐normally distributed variables were summarised as median with interquartile range [IQR], and Wilcoxon rank‐sum or Kruskal–Wallis (followed by Dunn's multiple comparison test) tests were used when examining differences between two or more than two groups, respectively. A Spearman's correlation test was used to examine the correlation between two continuous variables. Categorical data were described as number (frequency). Difference in proportions was analysed using Pearson's chi‐squared test or Fisher's exact test where appropriate.

Linear regression analysis was used to examine association between demographics and clinical parameters as exposure and log_10_‐transformed serum cytokine concentrations as outcome, as previously described.^27^ A bootstrap method repeated with 50 samples was used to derive robust confidence interval (CI). Results are presented using geometric mean (GM) and ratio of GM. GM and ratio of GM are defined as the antilog of the mean of a log_10_‐transformed value and the antilog of the regression coefficient, respectively. A *P*‐value of < 0.1 for association between potential confounders and both exposure and outcome variables in univariable analysis was used as a cut‐off for inclusion into a multivariable model. Serum cytokine concentrations were also categorised into binary variables, using their medians as cut‐off values. Values less than or equal to the median were considered as low serum cytokine concentrations, and values greater than the median were assigned as high serum cytokine concentrations. A *P*‐value < 0.05 was considered statistically significant.

## References

[1] Allanore Y , Simms R , Distler O *et al* Systemic sclerosis. Nat Rev Dis Primers 2015; 1: 15002. 27189141 10.1038/nrdp.2015.2

[2] Katsumoto TR , Whitfield ML , Connolly MK . The pathogenesis of systemic sclerosis. Annu Rev Pathol 2011; 6: 509–537. 21090968 10.1146/annurev-pathol-011110-130312

[3] van Bon L , Cossu M , Radstake TR . An update on an immune system that goes awry in systemic sclerosis. Curr Opin Rheumatol 2011; 23: 505–510. 21885976 10.1097/BOR.0b013e32834b0dac

[4] Scala E , Pallotta S , Frezzolini A *et al* Cytokine and chemokine levels in systemic sclerosis: relationship with cutaneous and internal organ involvement. Clin Exp Immunol 2004; 138: 540–546. 15544634 10.1111/j.1365-2249.2004.02642.x PMC1809238

[5] Dantas AT , Goncalves SM , de Almeida AR *et al* Reassessing the role of the active TGF‐β1 as a biomarker in systemic sclerosis: association of serum levels with clinical manifestations. Dis Markers 2016; 2016: 6064830. 27965520 10.1155/2016/6064830 PMC5124685

[6] Wu SP , Leng L , Feng Z *et al* Macrophage migration inhibitory factor promoter polymorphisms and the clinical expression of scleroderma. Arthritis Rheum 2006; 54: 3661–3669. 17075815 10.1002/art.22179

[7] Zhang L , Yan JW , Wang YJ *et al* Association of interleukin 1 family with systemic sclerosis. Inflammation 2014; 37: 1213–1220. 24531856 10.1007/s10753-014-9848-7

[8] Seymour HE , Worsley A , Smith JM , Thomas SH . Anti‐TNF agents for rheumatoid arthritis. Br J Clin Pharmacol 2001; 51: 201–208. 11298065 10.1046/j.1365-2125.2001.00321.x PMC2015031

[9] Dinarello CA . Interleukin 1 and interleukin 18 as mediators of inflammation and the aging process. Am J Clin Nutr 2006; 83: 447S–455S. 16470011 10.1093/ajcn/83.2.447S

[10] Sims JE , Smith DE . The IL‐1 family: regulators of immunity. Nat Rev Immunol 2010; 10: 89–102. 20081871 10.1038/nri2691

[11] Higgins GC , Wu Y , Postlethwaite AE . Intracellular IL‐1 receptor antagonist is elevated in human dermal fibroblasts that overexpress intracellular precursor IL‐1 alpha. J Immunol 1999; 163: 3969–3975. 10490999

[12] Kawaguchi Y , McCarthy SA , Watkins SC , Wright TM . Autocrine activation by interleukin 1alpha induces the fibrogenic phenotype of systemic sclerosis fibroblasts. J Rheumatol 2004; 31: 1946–1954. 15468358

[13] Umehara H , Kumagai S , Murakami M *et al* Enhanced production of interleukin‐1 and tumor necrosis factor alpha by cultured peripheral blood monocytes from patients with scleroderma. Arthritis Rheum 1990; 33: 893–897. 2363741 10.1002/art.1780330619

[14] Hutyrova B , Lukac J , Bosak V , Buc M , du Bois R , Petrek M . Interleukin 1alpha single‐nucleotide polymorphism associated with systemic sclerosis. J Rheumatol 2004; 31: 81–84. 14705223

[15] Duan H , Fleming J , Pritchard DK *et al* Combined analysis of monocyte and lymphocyte messenger RNA expression with serum protein profiles in patients with scleroderma. Arthritis Rheum 2008; 58: 1465–1474. 18438864 10.1002/art.23451

[16] Maekawa T , Jinnin M , Ohtsuki M , Ihn H . Serum levels of interleukin‐1α in patients with systemic sclerosis. J Dermatol 2013; 40: 98–101. 23078215 10.1111/1346-8138.12011

[17] Artlett CM , Sassi‐Gaha S , Rieger JL , Boesteanu AC , Feghali‐Bostwick CA , Katsikis PD . The inflammasome activating caspase 1 mediates fibrosis and myofibroblast differentiation in systemic sclerosis. Arthritis Rheum 2011; 63: 3563–3574. 21792841 10.1002/art.30568

[18] Gasse P , Mary C , Guenon I *et al* IL‐1R1/MyD88 signaling and the inflammasome are essential in pulmonary inflammation and fibrosis in mice. J Clin Invest 2007; 117: 3786–3799. 17992263 10.1172/JCI32285 PMC2066195

[19] Kantor TV , Friberg D , Medsger TA Jr , Buckingham RB , Whiteside TL . Cytokine production and serum levels in systemic sclerosis. Clin Immunol Immunopathol 1992; 65: 278–285. 1451330 10.1016/0090-1229(92)90158-k

[20] Hussein MR , Hassan HI , Hofny ER *et al* Alterations of mononuclear inflammatory cells, CD4/CD8+ T cells, interleukin 1β, and tumour necrosis factor α in the bronchoalveolar lavage fluid, peripheral blood, and skin of patients with systemic sclerosis. J Clin Pathol 2005; 58: 178–184. 15677539 10.1136/jcp.2004.019224 PMC1770564

[21] Henderson J , Bhattacharyya S , Varga J , O'Reilly S . Targeting TLRs and the inflammasome in systemic sclerosis. Pharmacol Ther 2018; 192: 163–169. 30081049 10.1016/j.pharmthera.2018.08.003

[22] Martinez‐Godinez MA , Cruz‐Dominguez MP , Jara LJ *et al* Expression of NLRP3 inflammasome, cytokines and vascular mediators in the skin of systemic sclerosis patients. Isr Med Assoc J 2015; 17: 5–10. 25739168

[23] Pan HF , Wang J , Leng RX , Li XP , Ye DQ . Interleukin‐18: friend or foe for systemic sclerosis? J Invest Dermatol 2011; 131: 2495. 21776011 10.1038/jid.2011.224

[24] Kim HJ , Song SB , Choi JM *et al* IL‐18 downregulates collagen production in human dermal fibroblasts via the ERK pathway. J Invest Dermatol 2010; 130: 706–715. 19865096 10.1038/jid.2009.302

[25] Zhang YJ , Zhang Q , Yang GJ *et al* Elevated serum levels of interleukin‐1β and interleukin‐33 in patients with systemic sclerosis in Chinese population. Z Rheumatol 2018; 77: 151–159. 27644954 10.1007/s00393-016-0202-3

[26] Jafari‐Nakhjavani MR , Abedi‐Azar S , Nejati B . Correlation of plasma interleukin‐18 concentration and severity of renal involvement and disease activity in systemic lupus erythematosus. J Nephropathol 2016; 5: 28–33. 27047807 10.15171/jnp.2016.05 PMC4790184

[27] Mende R , Vincent FB , Kandane‐Rathnayake R *et al* Analysis of serum interleukin (IL)‐1β and IL‐18 in systemic lupus erythematosus. Front Immunol 2018; 9: 1250. 29930551 10.3389/fimmu.2018.01250 PMC5999794

[28] Wu CY , Yang HY , Yao TC , Liu SH , Huang JL . Serum IL‐18 as biomarker in predicting long‐term renal outcome among pediatric‐onset systemic lupus erythematosus patients. Medicine (Baltimore) 2016; 95: e5037. 27749566 10.1097/MD.0000000000005037 PMC5059068

[29] Schmidt JA , Mizel SB , Cohen D , Green I . Interleukin 1, a potential regulator of fibroblast proliferation. J Immunol 1982; 128: 2177–2182. 6460819

[30] Coghlan JG , Denton CP , Grunig E *et al* Evidence‐based detection of pulmonary arterial hypertension in systemic sclerosis: the DETECT study. Ann Rheum Dis 2014; 73: 1340–1349. 23687283 10.1136/annrheumdis-2013-203301 PMC4078756

[31] Le Gouellec N , Duhamel A , Perez T *et al* Predictors of lung function test severity and outcome in systemic sclerosis‐associated interstitial lung disease. PLoS ONE 2017; 12: e0181692. 28763468 10.1371/journal.pone.0181692 PMC5538660

[32] Needleman BW , Wigley FM , Stair RW . Interleukin‐1, interleukin‐2, interleukin‐4, interleukin‐6, tumor necrosis factor alpha, and interferon‐gamma levels in sera from patients with scleroderma. Arthritis Rheum 1992; 35: 67–72. 1731816 10.1002/art.1780350111

[33] Akimoto S , Ishikawa O , Tamura T , Miyachi Y . Antineutrophil cytoplasmic autoantibodies in patients with systemic sclerosis. Br J Dermatol 1996; 134: 407–410. 8731661

[34] Esposito J , Brown Z , Stevens W *et al* The association of low complement with disease activity in systemic sclerosis: a prospective cohort study. Arthritis Res Ther 2016; 18: 246. 27770830 10.1186/s13075-016-1147-2 PMC5075219

[35] Font J , Ramos‐Casals M , Cervera R *et al* Antineutrophil cytoplasmic antibodies in primary Sjogren's syndrome: prevalence and clinical significance. Br J Rheumatol 1998; 37: 1287–1291. 9973150 10.1093/rheumatology/37.12.1287

[36] van den Hoogen F , Khanna D , Fransen J *et al* 2013 classification criteria for systemic sclerosis: an American College of Rheumatology/European League against Rheumatism collaborative initiative. Arthritis Rheum 2013; 65: 2737–2747. 24122180 10.1002/art.38098 PMC3930146

[37] Vincent FB , Lin E , Sahhar J *et al* Analysis of serum macrophage migration inhibitory factor and D‐dopachrome tautomerase in systemic sclerosis. Clin Transl Immunol 2018; 7: e1042. 10.1002/cti2.1042 PMC6283235 30546906

[38] Osthoff M , Ngian GS , Dean MM *et al* Potential role of the lectin pathway of complement in the pathogenesis and disease manifestations of systemic sclerosis: a case‐control and cohort study. Arthritis Res Ther 2014; 16: 480. 25403109 10.1186/s13075-014-0480-6 PMC4264552

[39] LeRoy EC , Black C , Fleischmajer R *et al* Scleroderma (systemic sclerosis): classification, subsets and pathogenesis. J Rheumatol 1988; 15: 202–205. 3361530

[40] Khanna D , Furst DE , Clements PJ *et al* Standardization of the modified Rodnan skin score for use in clinical trials of systemic sclerosis. J Scleroderma Relat Disord 2017; 2: 11–18. 28516167 10.5301/jsrd.5000231 PMC5431585

[41] Vincent FB , Nim HT , Lee JPW , Morand EF , Harris J . Effect of storage duration on cytokine stability in human serum and plasma. Cytokine 2018; 113: 453–457. 29909979 10.1016/j.cyto.2018.06.009

