# Supplementary material for: Analysis of serum interleukin(IL)‐1α, IL‐1β and IL‐18 in patients with systemic sclerosis
Source: Clin Transl Immunology. 2019 Apr 6;8(4):e1045. doi: 10.1002/cti2.1045 (PMC6451750; doi:10.1002/cti2.1045)
Supplement: Supplementary file 2 [file CTI2-8-e1045-s002.docx]

**Supplementary Table 1. Serum cytokine levels according to clinical manifestations in SSc.**

|  |  | **SSc patients (N = 105)** | | | | | | | |
| --- | --- | --- | --- | --- | --- | --- | --- | --- | --- |
|  | **n** | **Serum IL-1α (pg mL^-1^)** | |  | **Serum IL-1β (pg mL^-1^)** | |  | **Serum IL-18 (pg mL^-1^)** | |
|  |  | **Median [IQR]** | ***P*-value** | **n** | **Median [IQR]** | ***P*-value** | **n** | **Median [IQR]** | ***P*-value** |
| **Disease classification** |  |  | 0.68 |  |  | 0.82 |  |  | 0.29 |
| Limited | 70 | 11 [2, 29] |  | 82 | 6 [1, 18] |  | 80 | 270 [192, 372] |  |
| Diffuse | 17 | 9 [2, 23] |  | 23 | 7 [1, 12] |  | 23 | 245 [160, 324] |  |
| **PAH** |  |  | 0.74 |  |  | 0.79 |  |  | 0.31 |
| Absent | 82 | 11 [2, 29] |  | 100 | 7 [1, 17] |  | 98 | 262 [183, 360] |  |
| Present | 5 | 12 [6, 20] |  | 5 | 5 [4, 7] |  | 5 | 335 [221, 519] |  |
| **Pericardial effusion** |  |  | 0.89 |  |  | 0.27 |  |  | 0.95 |
| Absent | 84 | 11 [2, 29] |  | 100 | 6 [1, 17] |  | 98 | 258 [183, 362] |  |
| Present | 3 | 6 [2, 51] |  | 5 | 11 [9, 12] |  | 5 | 277 [276, 287] |  |
| **ILD** |  |  | 0.6 |  |  | 0.19 |  |  | 0.85 |
| Absent | 59 | 9 [2, 29] |  | 70 | 5 [1, 16] |  | 68 | 254 [186, 362] |  |
| Present | 28 | 13 [2, 35] |  | 35 | 11 [1, 18] |  | 35 | 276 [183, 356] |  |
| **Digital ulcers** |  |  | 0.13 |  |  | 0.35 |  |  | 0.25 |
| Absent | 71 | 11 [2, 29] |  | 86 | 7 [1, 17] |  | 85 | 276 [182, 362] |  |
| Present | 11 | 19 [13, 87] |  | 14 | 10 [5, 19] |  | 13 | 229 [198, 266] |  |
| **GAVE** |  |  | 0.9 |  |  | 0.9 |  |  | 0.21 |
| Absent | 78 | 11 [2, 29] |  | 96 | 7 [1, 17] |  | 94 | 261 [182, 356] |  |
| Present | 9 | 11 [2, 43] |  | 9 | 7 [1, 18] |  | 9 | 287 [229, 460] |  |
| **Reflux oesophagitis** |  |  | 0.43 |  |  | 0.85 |  |  | 0.27 |
| Absent | 37 | 12 [5, 29] |  | 46 | 8 [1, 17] |  | 44 | 241 [180, 324] |  |
| Present | 50 | 10 [2. 29] |  | 59 | 7 [1, 18] |  | 59 | 276 [198, 382] |  |
| **Oesophageal stricture** |  |  | 0.89 |  |  | 0.83 |  |  | 0.33 |
| Absent | 78 | 11 [2, 29] |  | 96 | 7 [1, 18] |  | 94 | 270 [193, 362] |  |
| Present | 9 | 14 [2, 20] |  | 9 | 6 [4, 9] |  | 9 | 209 [180, 339] |  |
| **Oesophageal dysmotility** |  |  | 0.25 |  |  | 0.63 |  |  | 0.47 |
| Absent | 83 | 11 [2, 33] |  | 100 | 7 [1, 17] |  | 98 | 270 [183, 362] |  |
| Present | 4 | 4 [2, 13] |  | 5 | 4 [1, 7] |  | 5 | 209 [201, 257] |  |
| **RP** |  |  | 0.52 |  |  | 0.29 |  |  | 0.36 |
| Absent | 14 | 7 [2, 35] |  | 15 | 5 [1, 12] |  | 15 | 305 [209, 363] |  |
| Present | 68 | 13 [2, 31] |  | 85 | 9 [1, 18] |  | 83 | 257 [180, 342] |  |
| **Calcinosis** |  |  | 0.62 |  |  | 0.34 |  |  | 0.45 |
| Absent | 64 | 12 [2, 35] |  | 77 | 7 [1, 16] |  | 75 | 258 [182, 341] |  |
| Present | 18 | 11 [2, 21] |  | 23 | 9 [1, 24] |  | 23 | 276 [180, 409] |  |
| **Synovitis** |  |  | 0.97 |  |  | 0.19 |  |  | 0.38 |
| Absent | 72 | 12 [2, 34] |  | 89 | 7 [1, 17] |  | 87 | 265 [191, 356] |  |
| Present | 10 | 9 [5, 29] |  | 11 | 11 [4, 27] |  | 11 | 198 [171, 363] |  |
| **Joint contracture** |  |  | 0.45 |  |  | 0.05 |  |  | 0.21 |
| Absent | 64 | 11 [2, 26] |  | 73 | 6 [1, 15] |  | 71 | 275 [191, 360] |  |
| Present | 18 | 16 [2, 43] |  | 27 | 14 [4, 19] |  | 27 | 238 [155, 341] |  |

GAVE: gastric antral vascular ectasia; ILD: interstitial lung fibrosis; PAH: pulmonary arterial hypertension; RP: Raynaud’s phenomenon; SSc: systemic sclerosis.

**Supplementary Table 2. Serum cytokine levels according to pulmonary and cardiac function tests in SSc.**

|  |  | **SSc patients (N = 105)** | | | | | | | |
| --- | --- | --- | --- | --- | --- | --- | --- | --- | --- |
|  | **n** | **Serum IL-1α (pg mL^-1^)** | |  | **Serum IL-1β (pg mL^-1^)** | |  | **Serum IL-18 (pg mL^-1^)** | |
|  |  | **Median [IQR]** | ***P*-value** | **n** | **Median [IQR]** | ***P*-value** | **n** | **Median [IQR]** | ***P*-value** |
| ***Pulmonary function tests*** |  |  |  |  |  |  |  |  |  |
| **FVC (%)** |  |  | 0.52 |  |  | 0.36 |  |  | 0.62 |
| Normal | 67 | 10 [2, 29] |  | 81 | 6 [1, 15] |  | 79 | 258 [182, 362] |  |
| Low (< 80%) | 19 | 17 [2, 35] |  | 23 | 12 [1, 20] |  | 23 | 265 [183, 362] |  |
| **FEV1 (%)** |  |  | 0.97 |  |  | 0.88 |  |  | 0.44 |
| Normal | 61 | 11 [2, 29] |  | 76 | 7 [1, 16] |  | 74 | 270 [197, 363] |  |
| Low (< 80%) | 25 | 11 [2, 33] |  | 28 | 9 [1, 18] |  | 28 | 245 [168, 341] |  |
| **DLCO (%)^#^** |  |  | 0.06 |  |  | 0.21 |  |  | 0.64 |
| Normal | 14 | 26 [4, 87] |  | 19 | 14 [1, 19] |  | 19 | 350 [160, 321] |  |
| Low (< 80%) | 64 | 11 [2. 26] |  | 76 | 7 [1, 17] |  | 74 | 262 [183, 342] |  |
| **KCO (%)^##^** |  |  | 0.04 |  |  | 0.2 |  |  | 0.06 |
| Normal | 15 | 24 [4, 89] |  | 18 | 15 [1, 20] |  | 18 | 206 [140, 305] |  |
| Low (< 80%) | 67 | 10 [2, 23] |  | 80 | 7 [1, 16] |  | 78 | 275 [197, 363] |  |
| ***Cardiac function tests*** |  |  |  |  |  |  |  |  |  |
| **Six-minute walk distance (m)** |  |  | 0.42 |  |  | 0.54 |  |  | 0.26 |
| Normal | 13 | 11 [4, 62] |  | 15 | 7 [1, 16] |  | 15 | 278 [155, 362] |  |
| Low (< 500m) | 11 | 15 [12, 28] |  | 14 | 6 [1, 22] |  | 14 | 349 [202, 363] |  |
| **LVEF (%)** |  |  | 0.62 |  |  | 0.49 |  |  | 0.62 |
| Normal | 57 | 12 [3, 35] |  | 72 | 9 [1, 18] |  | 70 | 248 [182, 321] |  |
| Low (< 55%) | 4 | 15 [2, 31] |  | 5 | 6 [1, 12] |  | 5 | 360 [170, 383] |  |
| **LV systolic function** |  |  | 0.78 |  |  | 0.54 |  |  | 0.34 |
| Normal | 68 | 12 [2, 28] |  | 84 | 7 [1, 17] |  | 82 | 254 [191, 342] |  |
| Abnormal | 5 | 20 [3, 33] |  | 5 | 7 [1, 9] |  | 5 | 180 [170, 276] |  |
| **RV dysfunction^*^** |  |  | 0.73 |  |  | 0.28 |  |  | 0.37 |
| Normal | 65 | 13 [2, 29] |  | 81 | 8 [1, 17] |  | 79 | 250 [182, 342] |  |
| Abnormal^###^ | 8 | 9 [2, 36] |  | 8 | 4 [1, 8] |  | 8 | 297 [200, 458] |  |
| **RV enlargement^*^** |  |  | 0.91 |  |  | 0.31 |  |  | 0.35 |
| Normal | 64 | 12 [2, 28] |  | 80 | 9 [1, 17] |  | 78 | 250 [182, 339] |  |
| Abnormal^###^ | 9 | 12 [2, 51] |  | 9 | 5 [1, 7] |  | 9 | 276 [221, 450] |  |
| **LA area (cm^2^)** |  |  | 0.61 |  |  | 0.42 |  |  | 0.06 |
| Normal | 36 | 13 [3, 31] |  | 43 | 8 [1, 18] |  | 43 | 230 [176, 321] |  |
| Abnormal (> 20 cm^2^) | 36 | 12 [2, 31] |  | 45 | 7 [1, 15] |  | 43 | 276 [198, 4089] |  |
| **TR gradient (mmHg)** |  |  | 0.56 |  |  | 0.52 |  |  | 0.36 |
| Normal | 53 | 14 [2, 33] |  | 66 | 9 [1, 18] |  | 65 | 250 [176, 339] |  |
| Abnormal (> 35 mmHg) | 9 | 12 [2, 20] |  | 11 | 5 [1, 18] |  | 11 | 266 [198, 522] |  |
| **TR velocity (m/s)** |  |  | 0.55 |  |  | 0.62 |  |  | 0.34 |
| Normal | 51 | 14 [2, 33] |  | 61 | 8 [1, 17] |  | 60 | 248 [165, 349] |  |
| Abnormal (> 2.8 m/s) | 9 | 12 [2, 20] |  | 13 | 7 [1, 26] |  | 13 | 275 [220, 335] |  |
| **sPAP (mmHg)** |  |  | 0.58 |  |  | 0.92 |  |  | 0.61 |
| Normal | 49 | 14 [2, 35] |  | 61 | 8 [1, 17] |  | 60 | 248 [177, 341] |  |
| Abnormal (> 40 mmHg) | 13 | 12 [2, 22] |  | 16 | 6 [1, 18] |  | 16 | 271 [189, 428] |  |

DLCO: corrected diffusing capacity of the lungs for carbon monoxide; FEV1: forced expiratory volume in one second; FVC: forced vital capacity; KCO: carbon monoxide transfer coefficient; LA: left atrial; LV: left ventricular; LVEF: left ventricular ejection fraction; RV: right ventricular; sPAP: systolic pulmonary arterial pressure; SSc: systemic sclerosis; TR: tricuspid regurgitation.

**^*^** 16 missing values.

**^#^** Corrected for haemoglobin and gender.

**^##^** DLCO corrected for lung volume.

^###^ Encompasses mild, moderate or severe.

**Supplementary Table 3. Serum cytokine levels according to laboratory markers in SSc.**

|  |  | **SSc patients (N = 105)** | | | | | | | |
| --- | --- | --- | --- | --- | --- | --- | --- | --- | --- |
|  | **n** | **Serum IL-1α (pg mL^-1^)** | |  | **Serum IL-1β (pg mL^-1^)** | |  | **Serum IL-18 (pg mL^-1^)** | |
|  |  | **Median [IQR]** | ***P*-value** | **n** | **Median [IQR]** | ***P*-value** | **n** | **Median [IQR]** | ***P*-value** |
| **ANA anti-centromere +ve** |  |  | 0.67 |  |  | 0.91 |  |  | 0.33 |
| No | 51 | 10 [2, 35] |  | 62 | 7 [1, 17] |  | 60 | 251 [180, 337] |  |
| Yes | 36 | 13 [2, 27] |  | 42 | 6 [1, 18] |  | 42 | 275 [197, 382] |  |
| **Anti-topoisomerase I Ab +ve** |  |  | 0.1 |  |  | 0.25 |  |  | 0.31 |
| No | 68 | 9 [2, 23] |  | 78 | 5 [1, 17] |  | 76 | 276 [192, 372] |  |
| Yes | 19 | 17 [4, 62] |  | 25 | 9 [4, 18] |  | 25 | 245 [182, 312] |  |
| **Anti-RNA polymerase III Ab +ve** |  |  | 0.4 |  |  | 0.99 |  |  | 0.56 |
| No | 76 | 11 [2, 26] |  | 93 | 7 [1, 17] |  | 91 | 266 [182, 362] |  |
| Yes | 9 | 35 [2, 51] |  | 9 | 7 [1, 20] |  | 9 | 229 [198, 276] |  |
| **CRP** |  |  | 0.23 |  |  | 0.63 |  |  | 0.33 |
| Normal | 55 | 15 [4, 39] |  | 66 | 7 [1, 17] |  | 65 | 245 [183, 321] |  |
| High (> 5 mg/L) | 22 | 12 [2, 22] |  | 29 | 9 [1, 18] |  | 28 | 281 [179, 373] |  |
| **ESR** |  |  | 0.92 |  |  | 0.09 |  |  | 0.67 |
| Normal | 71 | 13 [4, 35] |  | 84 | 9 [1, 18] |  | 83 | 245 [177, 341] |  |
| High (> 25 mm/h) | 4 | 15 [6, 41] |  | 9 | 12 [7, 30] |  | 9 | 258 [229, 287] |  |
| **Serum creatinine** |  |  | 0.05 |  |  | 0.06 |  |  | 0.03 |
| Normal or low | 74 | 14 [3, 35] |  | 92 | 9 [1, 18] |  | 90 | 250 [180, 335] |  |
| High (> 110 μmol/L) | 5 | 2 [2, 10] |  | 5 | 1 [1, 4] |  | 5 | 582 [341, 602] |  |

Ab: antibody; ANA: antinuclear antibodies; CRP: C-reactive protein; ESR: erythrocyte sedimentation rate; SSc: systemic sclerosis.

**Supplementary Table 4. Serum cytokine levels according to use of drugs in SSc.**

|  |  | **SSc patients (N = 105)** | | | | | | | |
| --- | --- | --- | --- | --- | --- | --- | --- | --- | --- |
|  | **n** | **Serum IL-1α (pg mL^-1^)** | |  | **Serum IL-1β (pg mL^-1^)** | |  | **Serum IL-18 (pg mL^-1^)** | |
|  |  | **Median [IQR]** | ***P*-value** | **n** | **Median [IQR]** | ***P*-value** | **n** | **Median [IQR]** | ***P*-value** |
| **Glucocorticoids** |  |  | 0.43 |  |  | 0.86 |  |  | 0.28 |
| Absent | 64 | 10 [2, 29] |  | 81 | 7 [1, 18] |  | 80 | 248 [182, 362] |  |
| Present | 23 | 13 [4, 35] |  | 24 | 6 [1, 15] |  | 23 | 294 [229, 356] |  |
| **PDE5 inhibitor** |  |  | 0.24 |  |  | 0.56 |  |  | 0.69 |
| Absent | 82 | 11 [2, 29] |  | 100 | 7 [1, 17] |  | 98 | 262 [183, 362] |  |
| Present | 5 | 20 [12, 22] |  | 5 | 7 [5, 15] |  | 5 | 276 [221, 335] |  |
| **ERA** |  |  | 0.32 |  |  | 0.85 |  |  | 0.71 |
| Absent | 83 | 11 [2, 29] |  | 100 | 7 [1, 17] |  | 98 | 262[183, 362] |  |
| Present | 4 | 16 [9, 67] |  | 5 | 5 [5, 7] |  | 5 | 266 [221, 335] |  |
| **Ca2^+^ channel antagonist** |  |  | 0.27 |  |  | 0.16 |  |  | 0.6 |
| Absent | 45 | 7 [2, 20] |  | 54 | 4 [1, 14] |  | 54 | 282 [193, 362] |  |
| Present | 42 | 15 [2, 35] |  | 51 | 9 [1, 19] |  | 49 | 250 [183, 360] |  |
| **Anticoagulant** |  |  | 0.23 |  |  | 0.52 |  |  | 0.71 |
| Absent | 80 | 11 [2, 26] |  | 98 | 6 [1, 17] |  | 96 | 262 [182, 361] |  |
| Present | 7 | 35 [2, 51] |  | 7 | 9 [1, 27] |  | 7 | 276 [198, 472] |  |
| **Anti-platelet agent** |  |  | 0.76 |  |  | 0.95 |  |  | 0.44 |
| Absent | 70 | 11 [2, 35] |  | 86 | 7 [1, 17] |  | 84 | 262[192, 348] |  |
| Present | 17 | 11 [3, 19] |  | 19 | 6 [1, 19] |  | 19 | 308 [180, 472] |  |
| **ACE inhibitor** |  |  | 0.49 |  |  | 0.72 |  |  | 0.08 |
| Absent | 78 | 11 [2, 28] |  | 94 | 6 [1, 18] |  | 93 | 276 [193, 362] |  |
| Present | 9 | 11 [9, 35] |  | 11 | 8 [1, 17] |  | 10 | 216 [170, 258] |  |
| **Angiotension II receptor blockers** |  |  | 0.63 |  |  | 0.38 |  |  | 0.51 |
| Absent | 72 | 11 [2, 31] |  | 88 | 7 [1, 18] |  | 87 | 257 [182, 362] |  |
| Present | 15 | 9 [2, 22] |  | 17 | 5 [1, 13] |  | 16 | 290 [192, 340] |  |
| **Beta blockers** |  |  | 0.52 |  |  | 0.3 |  |  | 0.46 |
| Absent | 82 | 11 [2, 29] |  | 100 | 7 [1, 18] |  | 98 | 262[(183, 360] |  |
| Present | 5 | 9 [2, 10] |  | 5 | 6 [1, 7] |  | 5 | 305 [229, 363] |  |

ACE: angiotensin converting enzyme; ERA: endothelin receptor antagonist; PDE5: phosphodiesterase 5; SSc: systemic sclerosis.

**Supplementary Table 5. SSc patient demographics and disease characteristics according to serum IL-18 median.**

|  | **Low serum IL-18 (n = 52)** | **High serum IL-18 (n = 51)** | ***P*-value** |
| --- | --- | --- | --- |
| ***Demographics*** |  |  |  |
| **Age (years)**, *mean (SD)* | 58 (14) | 62 (14) | 0.17 |
| **Female**, *n (%)* | 46 (88%) | 39 (76%) | 0.11 |
| **Ethnicity^*^**, *n (%)* |  |  | 0.45 |
| **Caucasian** | 41 (80%) | 43 (86%) |  |
| ***Clinical details*** |  |  |  |
| **Disease duration (years)**, *median [IQR]* | 13 [7, 19] | 12 [7, 19] | 0.94 |
| **Diffuse SSc**, *n (%)* | 13 (25%) | 10 (20%) | 0.51 |
| **Clinical manifestation** |  |  |  |
| PAH*, n (%)* | 2 (4%) | 3 (6%) | 0.68 |
| ILD*, n (%)* | 17 (33%) | 18 (35%) | 0.78 |
| Renal crisis*, n (%)* | 2 (4%) | 1 (2%) | 0.9 |
| Digital ulcers*, n (%)* | 9 (18%) | 4 (8%) | 0.23 |
| mRSS**,** *median [IQR]* | 6 (3, 9) | 5 (3, 8) | 0.28 |
| mRSS > 18*, n (%)* | 0 (0%) | 1 (2%) | 0.49 |
| RP*, n (%)* | 44 (88%) | 39 (81%) | 0.35 |
| Calcinosis*, n (%)* | 11 (22%) | 12 (25%) | 0.73 |
| Myositis*, n (%)* | 1 (2%) | 1 (2%) | 0.9 |
| Synovitis*, n (%)* | 6 (12%) | 5 (10%) | 0.9 |
| ***Pulmonary and cardiac function tests*** |  |  |  |
| **FVC**, *mean (SD)* | 94 (19) | 94 (18) | 0.9 |
| **DLCO**, *median [IQR]* | 62 [55, 74] | 55 [46, 73] | 0.29 |
| **DLCO low***, n (%)* | 38 (79%) | 36 (80%) | 0.9 |
| **KCO**, *mean (SD)* | 67 (17) | 61 (18) | 0.09 |
| **KCO low***, n (%)* | 38 (75%) | 40 (89%) | 0.11 |
| **LVEF (%)**, *median [IQR]* | 65 [60, 65] | 63 [55, 65] | 0.45 |
| **TR velocity (m/s)**, *median [IQR]* | 2 [2, 3] | 2 [2, 3] | 0.91 |
| ***Clinical Laboratory data*** |  |  |  |
| **ANA +ve***, n (%)* | 49 (96%) | 49 (96%) | 0.9 |
| **Anti-topoisomerase I***, n (%)* | 15 (29%) | 10 (20%) | 0.27 |
| **Anti-RNA polymerase III +ve***, n (%)* | 6 (12%) | 3 (6%) | 0.49 |
| **CRP (mg/L)**, *median [IQR]* | 3 [1, 6] | 4 [2, 7] | 0.13 |
| **ESR (mm/h)**, *median [IQR]* | 10 [5, 15] | 10 [5, 17] | 0.87 |
| **Creatinine (μmol/L)**, *median [IQR]* | 62 [51, 73] | 69 [59, 80] | < 0.01 |
| ***Treatment****, n (%)* |  |  |  |
| **Glucocorticoids** | 8 (15%) | 15 (29%) | 0.09 |
| **PDE5 inhibitor** | 2 (4%) | 3 (6%) | 0.68 |
| **ERA** | 2 (4%) | 3 (6%) | 0.68 |
| **Ca2^+^ channel antagonist** | 28 (54%) | 21 (41%) | 0.2 |
| **Anticoagulant** | 3 (6%) | 4 (8%) | 0.72 |
| **Anti-platelet agent** | 9 (17%) | 10 (20%) | 0.76 |
| **ACE inhibitor** | 8 (15%) | 2 (4%) | 0.09 |
| **Angiotensin II inhibitors** | 6 (12%) | 10 (20%) | 0.26 |
| **Beta blocker** | 2 (4%) | 3 (6%) | 0.68 |

^*^ 2 missing values.

DLCO: corrected diffusing capacity of the lungs for carbon monoxide; ERA: endothelin receptor antagonist; FVC: forced vital capacity; HAQ: health assessment questionnaire; ILD: interstitial lung disease; KCO: carbon monoxide transfer coefficient; LVEF: left ventricular ejection fraction; mRSS: modified Rodnan skin score; PAH: pulmonary arterial hypertension; PDE5: phosphodiesterase 5; RP: Raynaud’s phenomenon; SSc: systemic sclerosis; TR: tricuspid regurgitation.

**Supplementary Table 6. SSc patient demographics and disease characteristics according to serum IL1-β median.**

|  | **Low serum IL-1β (n = 53)** | **High serum IL-1β (n = 52)** | ***P*-value** |
| --- | --- | --- | --- |
| ***Demographics*** |  |  |  |
| **Age (years)**, *mean (SD)* | 61 (14) | 59 (14) | 0.61 |
| **Female**, *n (%)* | 42 (79%) | 45 (87%) | 0.32 |
| **Ethnicity^*^**, *n (%)* |  |  |  |
| **Caucasian** | 44 (83%) | 42 (84%) | 0.89 |
| ***Clinical details*** |  |  |  |
| **Disease duration (years)**, *median [IQR]* | 12 [6, 19] | 13 [8, 19] | 0.38 |
| **Diffuse SSc**, *n (%)* | 11 (21%) | 12 (23%) | 0.77 |
| **Clinical manifestation** |  |  |  |
| PAH*, n (%)* | 4 (8%) | 1 (2%) | 0.36 |
| ILD*, n (%)* | 12 (52%) | 22 (76%) | 0.07 |
| Renal crisis*, n (%)* | 2 (4%) | 2 (4%) | 0.9 |
| Digital ulcers*, n (%)* | 6 (12%) | 8 (16%) | 0.62 |
| mRSS**,** *median [IQR]* | 4 (3, 7) | 7 (4, 9) | 0.03 |
| mRSS > 18*, n (%)* | 1 (2%) | 0 (0%) | 0.49 |
| RP*, n (%)* | 40 (82%) | 45 (88%) | 0.36 |
| Calcinosis*, n (%)* | 11 (22%) | 12 (24%) | 0.9 |
| Myositis*, n (%)* | 1 (2%) | 1 (2%) | 0.9 |
| Synovitis*, n (%)* | 4 (8%) | 7 (14%) | 0.53 |
| ***Pulmonary and cardiac function tests*** |  |  |  |
| **FVC**, *mean (SD)* | 93 (19) | 94 (18) | 0.82 |
| **DLCO**, *median [IQR]* | 55 [44, 72] | 62 [55, 80] | 0.04 |
| **DLCO low***, n (%)* | 39 (85%) | 37 (76%) | 0.26 |
| **KCO**, *mean (SD)* | 60 (19) | 69 (15) | 0.02 |
| **KCO low***, n (%)* | 41 (85) | 39 (78) | 0.34 |
| **LVEF (%)**, *median [IQR]* | 60 (60, 65) | 65 (60, 65) | 0.37 |
| **TR velocity (m/s)**, *median [IQR]* | 2 [2, 3] | 3 [2, 3] | 0.05 |
| ***Clinical Laboratory data*** |  |  |  |
| **ANA +ve***, n (%)* | 49 (92%) | 51 (100%) | 0.12 |
| **Anti-topoisomerase I***, n (%)* | 10 (50%) | 15 (52%) | 0.91 |
| **Anti-RNA polymerase III +ve***, n (%)* | 4 (8%) | 5 (10%) | 0.9 |
| **CRP (mg/L)**, *median [IQR]* | 3 [2, 6] | 4 [1, 7] | 0.92 |
| **ESR (mm/h)**, *median [IQR]* | 11 [5, 17] | 10 [5, 20] | 0.76 |
| **Creatinine (μmol/L)**, *median [IQR]* | 66 [55, 86] | 64 [53, 72] | 0.38 |
| ***Treatment****, n (%)* |  |  |  |
| **Steroids** | 13 (25%) | 11 (21%) | 0.68 |
| **PDE5 inhibitor** | 3 (6%) | 2 (4%) | 0.9 |
| **ERA** | 4 (8%) | 1 (2%) | 0.36 |
| **Ca2^+^ channel antagonist** | 20 (38%) | 31 (60%) | 0.02 |
| **Anticoagulant** | 2 (4%) | 5 (10%) | 0.27 |
| **Anti-platelet agent** | 11 (21%) | 8 (15%) | 0.47 |
| **ACE inhibitor** | 4 (8%) | 7 (13%) | 0.36 |
| **Angiotensin II inhibitors** | 9 (17%) | 8 (15%) | 0.82 |
| **Beta blocker** | 3 (6%) | 2 (4%) | 0.9 |

^*^ 2 missing values.

DLCO: corrected diffusing capacity of the lungs for carbon monoxide; ERA: endothelin receptor antagonist; FVC: forced vital capacity; HAQ: health assessment questionnaire; ILD: interstitial lung disease; KCO: carbon monoxide transfer coefficient; LVEF: left ventricular ejection fraction; mRSS: modified Rodnan skin score; PAH: pulmonary arterial hypertension; PDE5: phosphodiesterase 5; RP: Raynaud’s phenomenon; SSc: systemic sclerosis; TR: tricuspid regurgitation.

**Supplementary Table 7. SSc patient demographics and disease characteristics according to serum IL-1α median.**

|  | **Low serum IL-1α (n = 44)** | **High serum IL-1α (n = 43)** | ***P*-value** |
| --- | --- | --- | --- |
| ***Demographics*** |  |  |  |
| **Age (years)**, *mean (SD)* | 62.5 (11.5) | 59.7 (13.7) | 0.30 |
| **Female**, *n (%)* | 38 (86%) | 36 (84%) | 0.73 |
| **Ethnicity^*^**, *n (%)* |  |  |  |
| **Caucasian** | 37 (84%) | 34 (81%) | 0.7 |
| ***Clinical details*** |  |  |  |
| **Disease duration (years)**, *median [IQR]* | 12.8 (5.9, 19.5) | 11.7 (5.2, 19.2) | 0.79 |
| **Diffuse SSc**, *n (%)* | 10 (23%) | 7 (16%) | 0.45 |
| **Clinical manifestation** |  |  |  |
| PAH*, n (%)* | 2 (5%) | 3 (7%) | 0.68 |
| ILD*, n (%)* | 11 (25%) | 17 (40%) | 0.15 |
| Renal crisis*, n (%)* | 2 (5%) | 2 (5%) | 0.9 |
| Digital ulcers*, n (%)* | 2 (5%) | 9 (21%) | 0.05 |
| mRSS**,** *median [IQR]* | 4.0 (3.0, 8.0) | 5.0 (3.0, 8.0) | 0.68 |
| mRSS > 18*, n (%)* | 1 (3%) | 0 (0%) | 0.48 |
| RP*, n (%)* | 31 (79%) | 37 (86%) | 0.43 |
| Calcinosis*, n (%)* | 9 (23%) | 9 (21%) | 0.81 |
| Myositis*, n (%)* | 0 (0%) | 2 (5%) | 0.24 |
| Synovitis*, n (%)* | 6 (15%) | 4 (9%) | 0.51 |
| ***Pulmonary and cardiac function tests*** |  |  |  |
| **FVC**, *mean (SD)* | 93.5 (18.5) | 94.2 (20.3) | 0.87 |
| **DLCO**, *median [IQR]* | 54.8 (46.7, 71.4) | 59.9 (44.2, 77.4) | 0.51 |
| **DLCO low***, n (%)* | 33 (87%) | 31 (78%) | 0.2 |
| **KCO**, *mean (SD)* | 60.0 (17.9) | 66.5 (17.9) | 0.1 |
| **KCO low***, n (%)* | 37 (90%) | 30 (73%) | 0.08 |
| **LVEF (%)**, *median [IQR]* | 62.5 (60.0, 65.0) | 65.0 (60.0, 65.0) | 0.38 |
| **TR velocity (m/s)**, *median [IQR]* | 2.4 (2.2, 2.8) | 2.5 (2.3, 2.7) | 0.29 |
| ***Clinical Laboratory data*** |  |  |  |
| **ANA +ve***, n (%)* | 40 (91%) | 43 (100%) | 0.12 |
| **Anti-topoisomerase I***, n (%)* | 7 (16%) | 12 (28%) | 0.18 |
| **Anti-RNA polymerase III +ve***, n (%)* | 4 (10%) | 5 (12%) | 0.9 |
| **CRP (mg/L)**, *median [IQR]* | 3.2 (1.6, 6.0) | 2.8 (1.4, 6.0) | 0.44 |
| **ESR (mm/h)**, *median [IQR]* | 9.5 (5.0, 17.0) | 10.0 (5.0, 15.0) | 0.69 |
| **Creatinine (μmol/L)**, *median [IQR]* | 64.5 (54.5, 92.5) | 65.0 (53.0, 75.0) | 0.62 |
| ***Treatment****, n (%)* |  |  |  |
| **Steroids** | 10 (23%) | 13 (30%) | 0.43 |
| **PDE5 inhibitor** | 1 (2%) | 4 (9%) | 0.2 |
| **ERA** | 1 (2%) | 3 (7%) | 0.36 |
| **Ca2^+^ channel antagonist** | 17 (39%) | 25 (58%) | 0.07 |
| **Anticoagulant** | 2 (5%) | 5 (12%) | 0.27 |
| **Anti-platelet agent** | 8 (18%) | 9 (21%) | 0.75 |
| **ACE inhibitor** | 5 (11%) | 4 (9%) | 0.9 |
| **Angiotensin II inhibitors** | 8 (18%) | 7 (16%) | 0.81 |
| **Beta blocker** | 4 (9%) | 1 (2%) | 0.36 |

^*^ 2 missing values.

DLCO: corrected diffusing capacity of the lungs for carbon monoxide; ERA: endothelin receptor antagonist; FVC: forced vital capacity; HAQ: health assessment questionnaire; ILD: interstitial lung disease; KCO: carbon monoxide transfer coefficient; LVEF: left ventricular ejection fraction; mRSS: modified Rodnan skin score; PAH: pulmonary arterial hypertension; PDE5: phosphodiesterase 5; RP: Raynaud’s phenomenon; SSc: systemic sclerosis; TR: tricuspid regurgitation.
