# Supplementary material for: Analysis of serum interleukin(IL)‐1α, IL‐1β and IL‐18 in patients with systemic sclerosis
Source: Clin Transl Immunology. 2019 Apr 6;8(4):e1045. doi: 10.1002/cti2.1045 (PMC6451750; doi:10.1002/cti2.1045)
Supplement: Supplementary file 3 [file CTI2-8-e1045-s003.docx]

**Supplementary Figure 1. Correlation between serum IL-1α, IL-1β and IL-18 concentrations in SSc and HC.**

Correlation between serum concentrations of IL-1α and IL-1β in **(a)** SSc (N = 87) and **(b)** HC (N = 42). Correlation between serum concentrations of IL-1α and IL-18 in **(c)** SSc (N = 85) and **(d)** HC (N = 42). Correlation between serum concentrations of IL-1β and IL-18 in **(e)** SSc (N = 103) and **(f)** HC (N = 47). *Panels a-f*: Spearman’s correlation test was used to examine the correlation between two continuous variables.
